# Relationship between estimated glomerular filtration rate, albuminuria, and oxidant status in the Japanese population

**DOI:** 10.1186/1471-2369-14-191

**Published:** 2013-09-09

**Authors:** Yuko Ishizaka, Minoru Yamakado, Akiko Toda, Mizuki Tani, Nobukazu Ishizaka

**Affiliations:** 1 Center for Multiphasic Health Testing and Services, Mitsui Memorial Hospital, Tokyo, Japan; 2 Department of Cardiology, Osaka Medical College, 2-7 Daigakumachi, Takatsuki-shi, 569-8686 Osaka, Japan

**Keywords:** Oxidative stress, Antioxidant potentials, Chronic kidney disease

## Abstract

**Background:**

In the general population, reported levels of oxidative stress and antioxidant potential seem to vary. The aim of this study was to investigate the levels of oxidant status markers in relation to estimated glomerular filtration rate (eGFR) and albuminuria in Japanese population.

**Methods:**

Data were analyzed from 8335 individuals who underwent a general health screening test. For the estimation of albuminuria, urinary albumin-to-creatinine ratio (UAER) was calculated. Oxidant status was determined by assessing derivatives of reactive oxygen metabolites (d-ROMs) and biological antioxidant potential (BAP).

**Results:**

After adjusting for age, high blood pressure, depressor agent use, CRP, smoking status, multivariate logistic regression analysis showed that the lowest eGFR quartile was associated negatively with the top d-ROM quartile in men (odds ratio 0.78 [95% CI 0.62-0.98, P = 0.034]) and the highest UAER was associated with the top d-ROM in men (odds ratio 1.68) [95% CI 1.35-2.10, P < 0.001]. In addition, both the first eGFR quartile and the fourth UAER quartile showed significant positive association with low BAP levels in men, but not in women.

**Conclusions:**

Among men who underwent general health screening, lower eGFR and increased albuminuria was negatively and positively, respectively, associated with higher oxidative stress levels, whereas both conditions were positively associated with lower antioxidant potential levels.

## Background

The prevalence of chronic kidney disease (CKD) in the general population has been predicted to be between 6.8% and more than one-fifth [1-3], depending on the age and ethnicity of the target population. Two conditions have been utilized to diagnose CKD; low estimated glomerular filtration rate (eGFR) and high albuminuria, although the presence or absence of CKD is sometimes defined solely on the basis of eGFR data. Considering that both low eGFR and high albuminuria are both associated with cardiovascular morbidity, mortality [4], and end-stage renal disease [5], CKD is recognized as a major public health problem.

Several previous studies have suggested that oxidative stress is associated with and involved in the pathogenesis of CKD and its complications [6,7]. Oxidative stress in CKD is presumed to be caused by a combination of increased production of reactive oxygen species [8] and impaired antioxidant capacity [9], and an enhancement of oxidative stress may also occur alongside the two frequently associated conditions, hypertension and diabetes. The findings that antioxidant therapy improved vascular function [10] and disease pathologies [11] may further support this notion.

These observations suggest that monitoring oxidative stress and antioxidant potential in vivo will provide crucial information for avoiding the progression of CKD and life-threatening events related to CKD; however, most of the methods for measuring these parameters may not be applicable to screening a low-risk general population of large size owing to the complexity of the analytical methodology, and cost. Recently, automated methods for the measurement of derivatives of reactive oxygen metabolites (d-ROMs) and biological antioxidant potential (BAP) have become available [12,13]. To this end, we assessed whether CKD, low eGFR and high albuminuria, are associated with enhanced oxidative stress and antioxidant potential in individuals who underwent general health screening.

## Methods

### Study population

The study was approved by the Ethics Committees of Mitsui Memorial Hospital and Osaka Medical College. This study was following the Guideline for Epidemiological Studies; therefore, written informed consent was not taken from each potential study participants. The study population comprised 8335 individuals aged ≥ 20 years (2958 women, 5377 men) who underwent a general health screening test and including the measurement of serum markers for oxidant status as well as blood pressure, renal function and extent of proteinuria (urinary excretion and creatinine excretion) between 2008 and 2012. In Japan, regular health check-ups for employees are mandated by law and, therefore, the majority of the study subjects did not have serious health problems. Blood pressure was measured after about 10 min of rest by an automated sphygmomanometer. In the current study, high blood pressure was defined to be present when systolic blood pressure was ≥ 140 mmHg and/or diastolic blood pressure was ≥ 90 mmHg.

### Laboratory analysis

Blood samples were taken from subjects after an overnight fast. Blood chemistry was performed as described previously [14]. Both the oxidative stress level and antioxidant properties in serum were determined by an automated method as described previously. In brief, the oxidative stress level was assessed via a d-ROMs test (Diacron s.r.l., Italy) [15,16] and antioxidant potential was assessed via a BAP test (Diacron s.r.l.) [17,18] using an automatic analyzer. When serum sample is dissolved in an acidic buffer of d-ROM test, the hydroperoxides react with the transition metal ions that are liberated from the protein in the acidic medium, resulting in an conversion to proxy and peroxyl radicals, which are able to oxidize N,N-diethyl-para-phenylenediamine which can eventually be detected spectrophotometrically [12,19]. For the determination of antioxidant potential, BAP test measures of antioxidants as agents that can reduce iron from ferric (Fe^3+^) to ferrous form (Fe^2+^), and chromatic change of this reaction will be measured photometrically [20]. Measurements obtained in the d-ROMs and BAP tests were expressed as Carr units [21] and μmol/L, respectively. eGFR was calculated on the basis of the new Japanese coefficient-modified Modification of Diet in Renal disease (MDRD) study equation [22]: eGFR (mL/min/1.73 m^2^) = 194 × (serum creatinine)^-1.094^ × (age)^-0.287^ (× 0.739, when female). For the diagnosis of albuminuria, spot urine samples were collected and calculated on the basis of 1 g of urinary creatinine; increased albuminuria was defined to be present when the urinary albumin-to-creatinine ratio (urinary albumin excretion ratio, UAER) was ≥ 30 (mg/g·creatinine), and micro- and macro-albuminuria was defined when UAER (mg/g ·creatinine) was 30-300 and ≥ 300, respectively.

### Statistical analysis

Data analysis was performed by using IBM SPSS statistics version 21.0 (SPSS, Chicago, IL). Continuous variables are expressed as the mean ± standard deviation or median and interquartile range, and dichotomous variables are expressed as number and percentage, unless stated otherwise. Differences between groups were calculated by analysis of variance (ANOVA), Dunnett’s post hoc analysis, and χ^2^ tests, where appropriate. Correlations between variables tested were assessed by Spearman's correlation coefficient. In the logistic regression analysis, age, high blood pressure, antihypertensive drug use, smoking (former, current), the quartiles of GFR and UAER were used as independent variables. A value of p < 0.05 was taken to be statistically significant.

## Results

### Characteristics of Individuals

**Figure 1:**
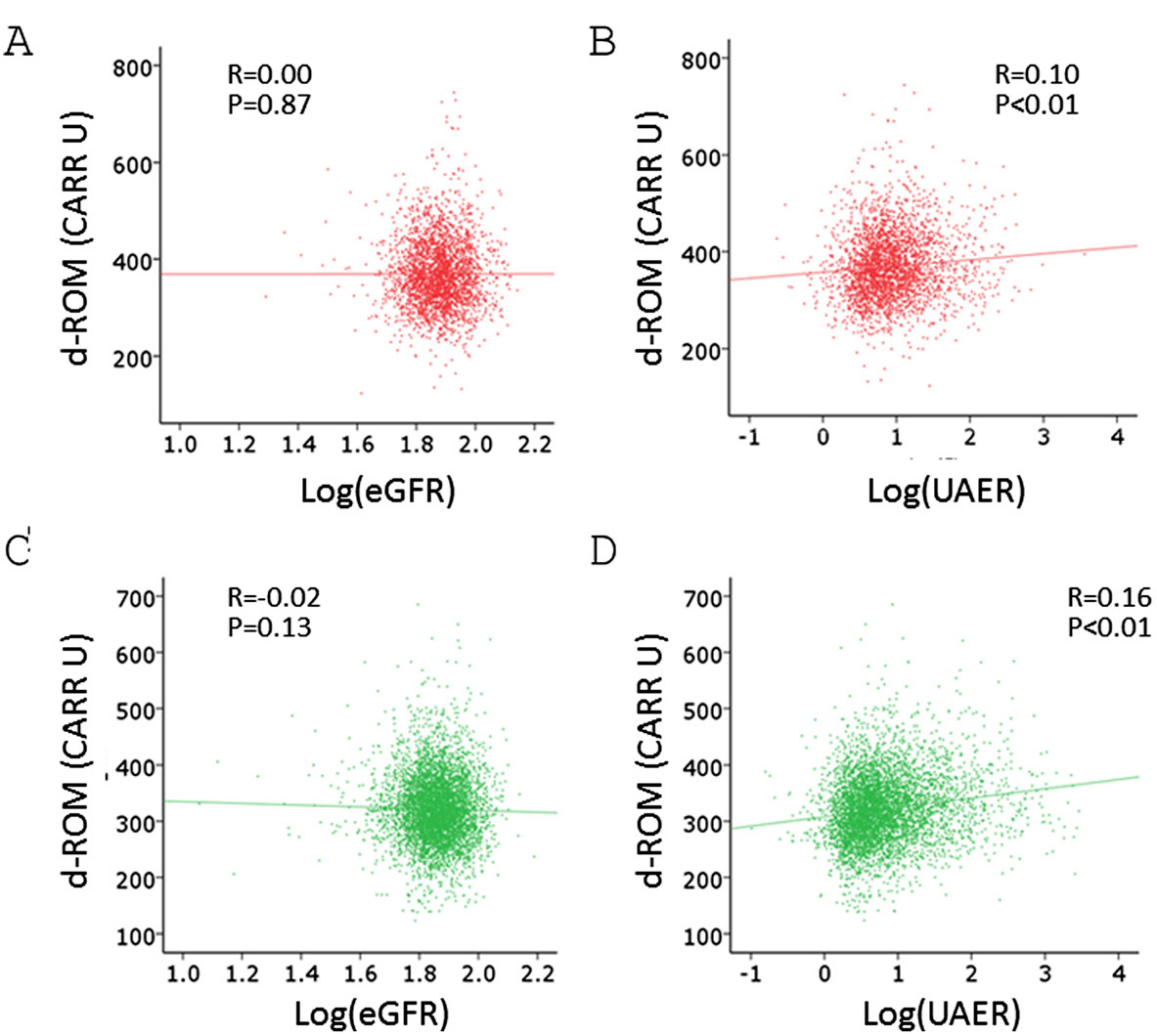
**Correlation between log(eGFR), log(UAER) and d-ROM levels.** Spearman’s correlation analysis was performed. **A**. Correlation between log(eGFR) and d-ROM in women. **B**. Correlation between log(UAER) and d-ROM in women. **C**. Correlation between log(eGFR) and d-ROM in men. **D**. Correlation between log(UAER) and d-ROM in men.

**Figure 2:**
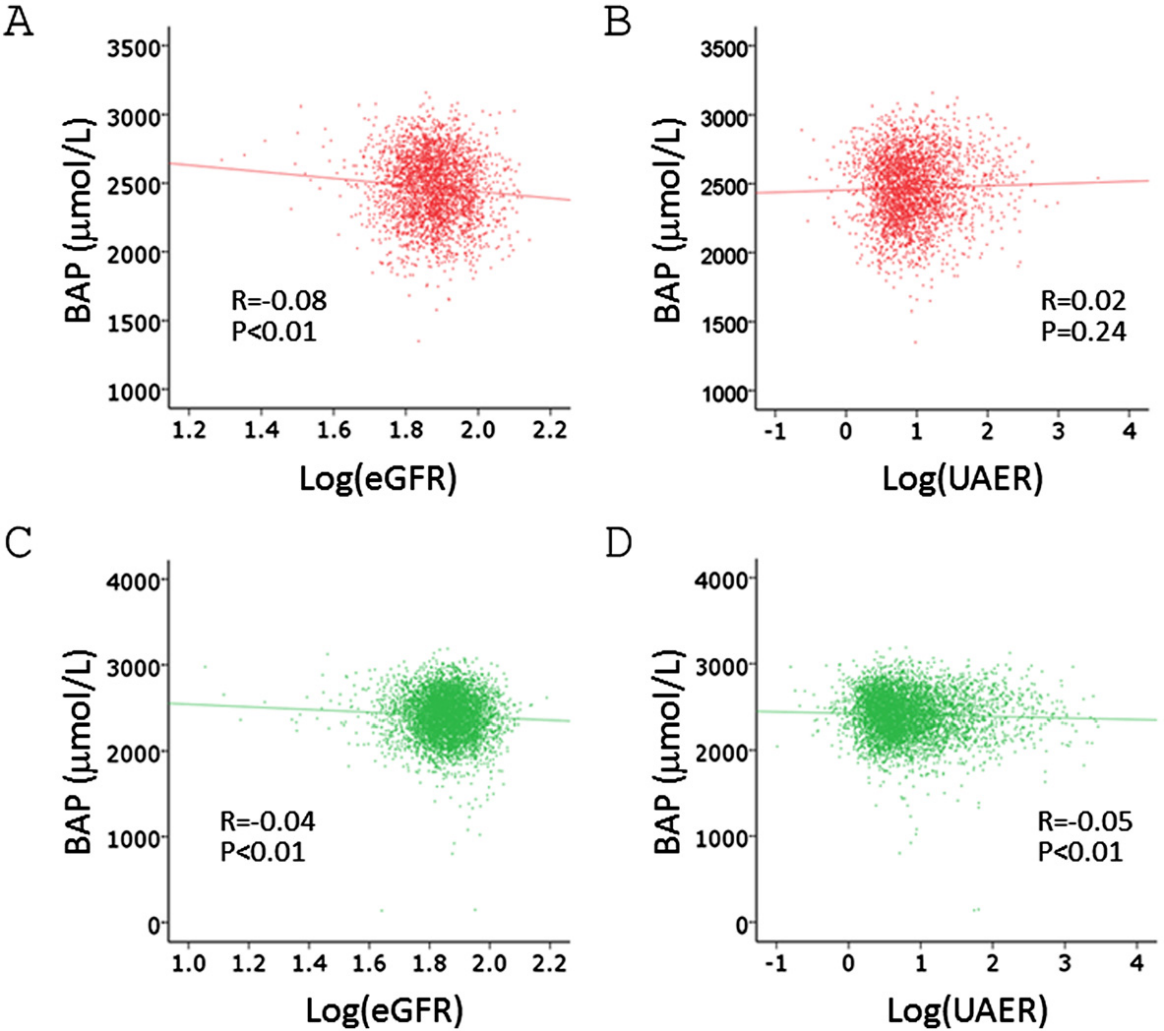
**Correlation between log(eGFR), log(UAER) and BAP levels.** Spearman’s correlation analysis was performed. **A**. Correlation between log(eGFR) and BAP in women. **B**. Correlation between log(UAER) and d-BAP in women. **C**. Correlation between log(eGFR) and d-BAP in men. **D**. Correlation between log(UAER) and d-BAP in men.

The mean age of the study subjects was 59.7 ± 10.4 years, and did not differ significantly between women and men (Table 1). Both d-ROM and BAP values were significantly higher in women than in men. By Spearman’s test, d-ROM and BAP showed positive, albeit very weak, correlation in either gender (Table 2). Correlation between log(eGFR) and d-ROM was not significant in either gender, and that between log(UAER) and BAP was not significant in women (Figures 1 and 2, Table 2).

**Table 1:** Baseline characteristics

	**Whole subjects**						**Women**						**Men**						**P value**
No. of subjects	8335						2958						5377						0.862
Age, years	59.7	±	10.4				59.7	±	10.1				59.6	±	10.5				<0.001
BMI, kg/m^2^	23.2	±	3.3				21.8	±	3.3				24.1	±	3.1				<0.001
Waist circumference, cm	83.3	±	9.4				78.6	±	9.5				85.9	±	8.3				<0.001
Systolic blood pressure, mmHg	125.0	±	17.7				120.4	±	18.7				127.6	±	16.6				<0.001
Diastolic blood pressure, mmHg	78.3	±	10.7				74.4	±	10.8				80.6	±	10.0				<0.001
Heart rate, bpm	62.5	±	9.2				63.2	±	8.9				62.2	±	9.4				<0.001
Laboratory data																			
LDL Cholesterol, mg/dL	124.0	±	29.8				127.2	±	31.1				122.3	±	28.9				<0.001
HDL Cholesterol, mg/dL	62.0	±	15.4				69.7	±	15.1				57.8	±	13.8				<0.001
Triglycerides, mg/dL	118	±	76.6				91	±	46.7				132	±	85.4				<0.001
C-reactive protein, mg/dL	0.04	(	0.02	-	0.08	)	0.03	(	0.01	-	0.06	)	0.04	(	0.02	-	0.09	)	<0.001
FBS, mg/dL	101.7	±	19.1				96.1	±	17.1				104.7	±	19.5				<0.001
HbA1C,%	5.5	±	0.6				5.4	±	0.5				5.5	±	0.6				<0.001
BUN, U/L	14.9	±	3.7				14.4	±	3.6				15.1	±	3.8				<0.001
Creatinine, mg/dL	0.8	±	0.2				0.6	±	0.1				0.9	±	0.2				<0.001
eGFR, mL/min/1.73 m^2^	73.5	(	65.1	-	82.6	)	75.6	(	66.5	-	85.1	)	72.3	(	64.5	-	81.4	)	<0.001
UAER, mg/g Cr	5.8	(	3.3	-	12.8	)	7.3	(	4.3	-	14.5	)	5.0	(	2.9	-	11.5	)	<0.001
Oxidative stress markers																			
d-ROM, CARR U	338	±	66				369	±	68				321	±	59				<0.001
BAP, μmol/L	2430	±	251				2468	±	239				2408	±	255				<0.001
Smoking status																			
Never (%)	4069	(	48.8	)			2430	(	82.2	)			1639	(	30.5	)			<0.001
Former (%)	3118	(	37.4	)			355	(	12.0	)			2463	(	51.4	)			
Current (%)	1148	(	13.8	)			173	(	5.8	)			975	(	18.1	)			
Medication																			
Antihypertensive (%)	1815	(	21.8	)			457	(	15.4	)			1358	(	25.3	)			<0.001
Glucose lowering (%)	355	(	4.3	)			69	(	2.3	)			286	(	5.3	)			<0.001
Lipid lowering (%)	871	(	10.4	)			339	(	11.5	)			532	(	9.9	)			0.025

Triglycerides, C-reactive protein, and UAER were expressed as median (interquartile range).

**Table 2:** Spearman’s correlation coefficients with d-ROMs and BAP

	**d-ROM**		**BAP**	
	**r**	**P value**	**r**	**P value**
Whole subjects				
d-ROM	-		0.19	<0.001
BAP	0.19	<0.001	-	
Age	0.10	<0.001	0.00	0.813
BSA	−0.26	<0.001	−0.11	<0.001
Serum creatinine	−0.26	<0.001	−0.03	0.002
C-reactive protein	0.31	<0.001	−0.04	<0.001
eGFR	0.03	0.013	−0.04	0.001
UAER	0.19	<0.001	−0.01	0.285
Women				
d-ROM	-		0.16	<0.001
BAP	0.16	<0.001	-	
Age	0.10	<0.001	0.03	0.112
BSA	0.08	<0.001	−0.01	0.609
Serum creatinine	−0.04	0.036	0.08	<0.001
C-reactive protein	0.41	<0.001	−0.01	0.651
eGFR	0.00	0.877	−0.08	<0.001
UAER	0.10	<0.001	0.02	0.267
Men				
d-ROM	-		0.17	<0.001
BAP	0.17	<0.001	-	
Age	0.10	<0.001	−0.02	0.177
BSA	−0.03	0.012	−0.05	<0.001
Serum creatinine	−0.01	0.378	0.05	<0.001
C-reactive protein	0.38	<0.001	−0.03	0.017
eGFR	−0.02	0.131	−0.04	0.006
UAER	0.16	<0.001	−0.05	<0.001

### Multivariate analysis

**Figure 3:**
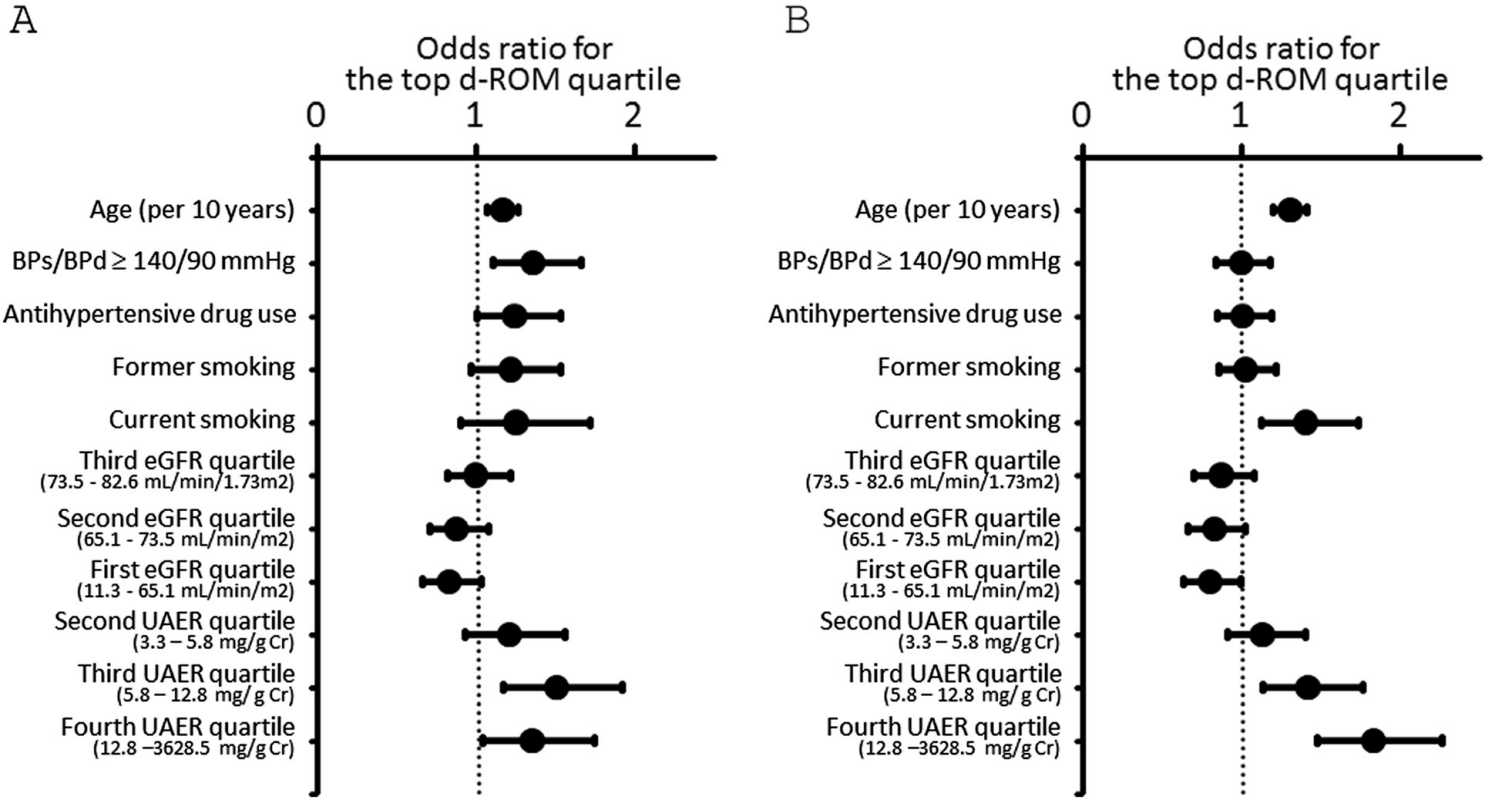
**Logistic regression analysis examining the association between various parameters and the top d-ROM quartile. A**. Association of various parameters with the top d-ROM quartile in women. **B**. Association of various parameters with the top d-ROM quartile in men. For former and current smoking, never smoking was used as reference. For eGFR and UAER quartiles, the fourth eGFR quartile and the first UAER quartile, respectively, was used as reference.

**Figure 4:**
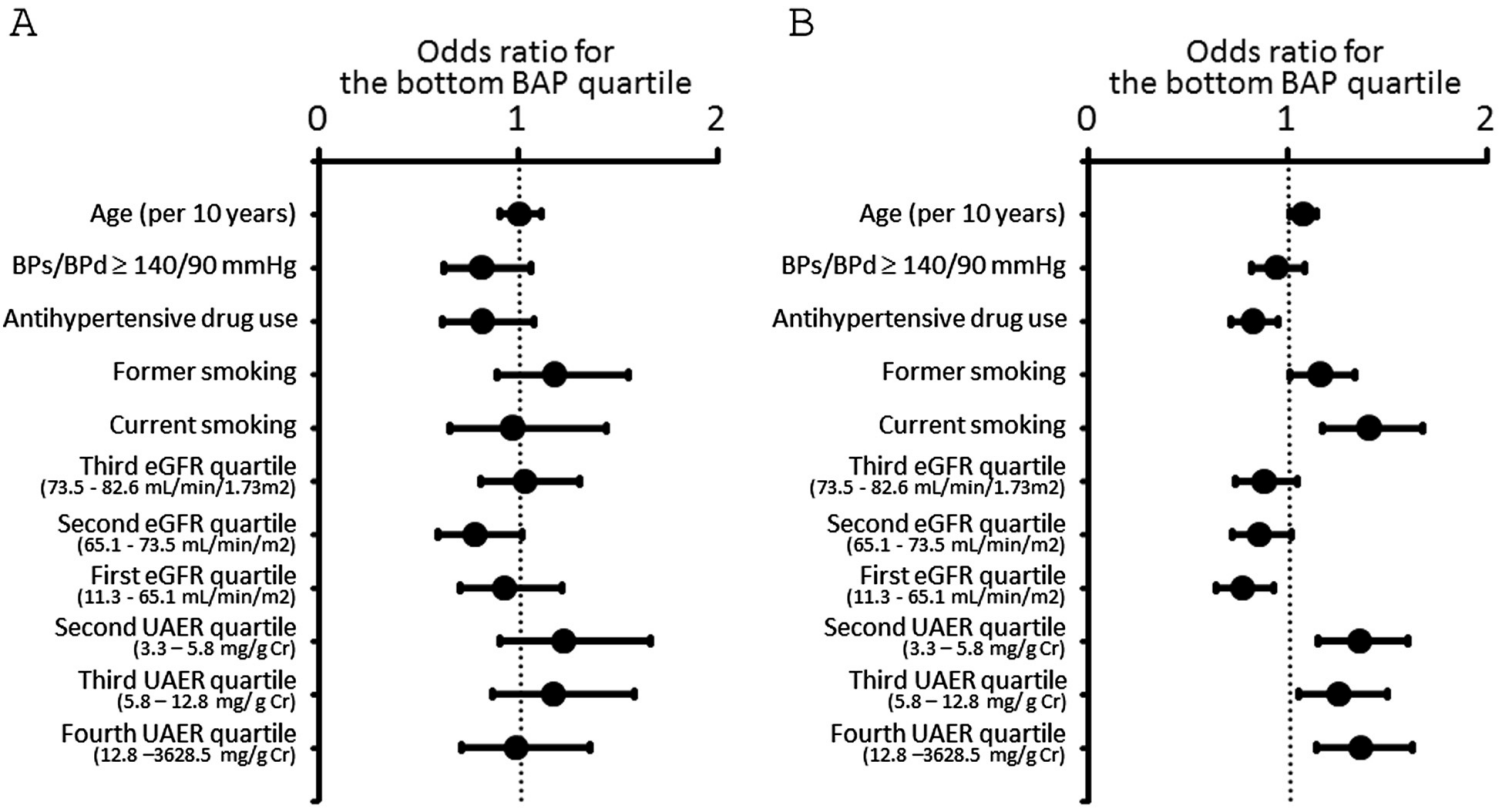
**Logistic regression analysis examining the association between various parameters and the bottom BAP quartile. (A)** Association of various parameters with the bottom BAP quartile in women. **(B)** Association of various parameters with the bottom BAP quartile in men. For former and current smoking, never smoking was used as reference. For eGFR and UAER quartiles, the fourth eGFR quartile and the first UAER quartile, respectively, was used as reference.

We investigated the association between oxidant status markers and various variables including eGFR quartiles (the highest eGFR quartile was used as the reference), UAER quartiles (the lowest UAER quartile was used as the reference), age, high blood pressure, antihypertensive drugs use, and smoking status (Figures 3 and 4). In this model, the third and fourth UAER quartiles showed significant association with top d-ROM quartile (≥ 376 Carr units) in both genders. In addition, the second through fourth UAER quartiles were associated with the bottom BAP quartile (< 2263 μmol/L) in men. On the other hand, in men, the first eGFR quartile was negatively associated with the top d-ROM quartile or with the bottom BAP quartile.

When C-reactive protein (CRP) was added as one of the covariates of the above statistical model, odds ratio of the lowest eGFR or the highest UAER quartile for the top d-ROM quartile or for the bottom BAP quartile was essentially unchanged, except the loss of significant association between the fourth UAER quartile and the top d-ROM quartile in women (Table 3). In this model, CRP was found to be significantly associated with the top d-ROM quartile in both genders, whereas it did not show significant association with the bottom BAP quartile in either gender.

**Table 3:** Odds ratio for the top d-ROM or the bottom BAP quartile before and after including CRP levels as one of the covariates

**Dependent variable**	**Independent variable**	**Odd ratios**	**(**	**95%**			**)**	**P value**
The top d-ROM quartile	Women							
	Model 1							
	The first eGFR quartile	0.83	(	0.66	-	1.04	)	0.100
	The fourth UAER quartile	1.34	(	1.04	-	1.75	)	0.023
	Model 2							
	The first eGFR quartile	0.81	(	0.64	-	1.02	)	0.079
	The fourth UAER quartile	1.16	(	0.18	-	1.52	)	0.268
	Men							
	Model 1							
	The first eGFR quartile	0.80	(	0.64	-	1.00	)	0.045
	The fourth UAER quartile	1.83	(	1.48	-	2.26	)	<0.001
	Model 2							
	The first eGFR quartile	0.78	(	0.62	-	0.98	)	0.034
	The fourth UAER quartile	1.68	(	1.35	-	2.10	)	<0.001
The bottom BAP quartile	Women							
	Model 1							
	The first eGFR quartile	0.93	(	0.70	-	1.22	)	0.589
	The fourth UAER quartile	0.99	(	0.72	-	1.36	)	0.927
	Model 2							
	The first eGFR quartile	0.93	(	0.71	-	1.23	)	0.617
	The fourth UAER quartile	1.00	(	0.73	-	1.38	)	0.994
	Men							
	Model 1							
	The first eGFR quartile	0.77	(	0.64	-	0.93	)	0.005
	The fourth UAER quartile	1.36	(	1.14	-	1.63	)	0.001
	Model 2							
	The first eGFR quartile	0.77	(	0.64	-	0.93	)	0.005
	The fourth UAER quartile	1.37	(	1.15	-	1.64	)	<0.001

In model 1, variables used in Figures 2 and 3 were used as dependent variables (age, high blood pressure, hypertensive drug use, smoking status, eGFR quartiles, UAER quartiles). In model 2, CRP levels were added as one of the independent variables to model 1.

**Figure 5:**
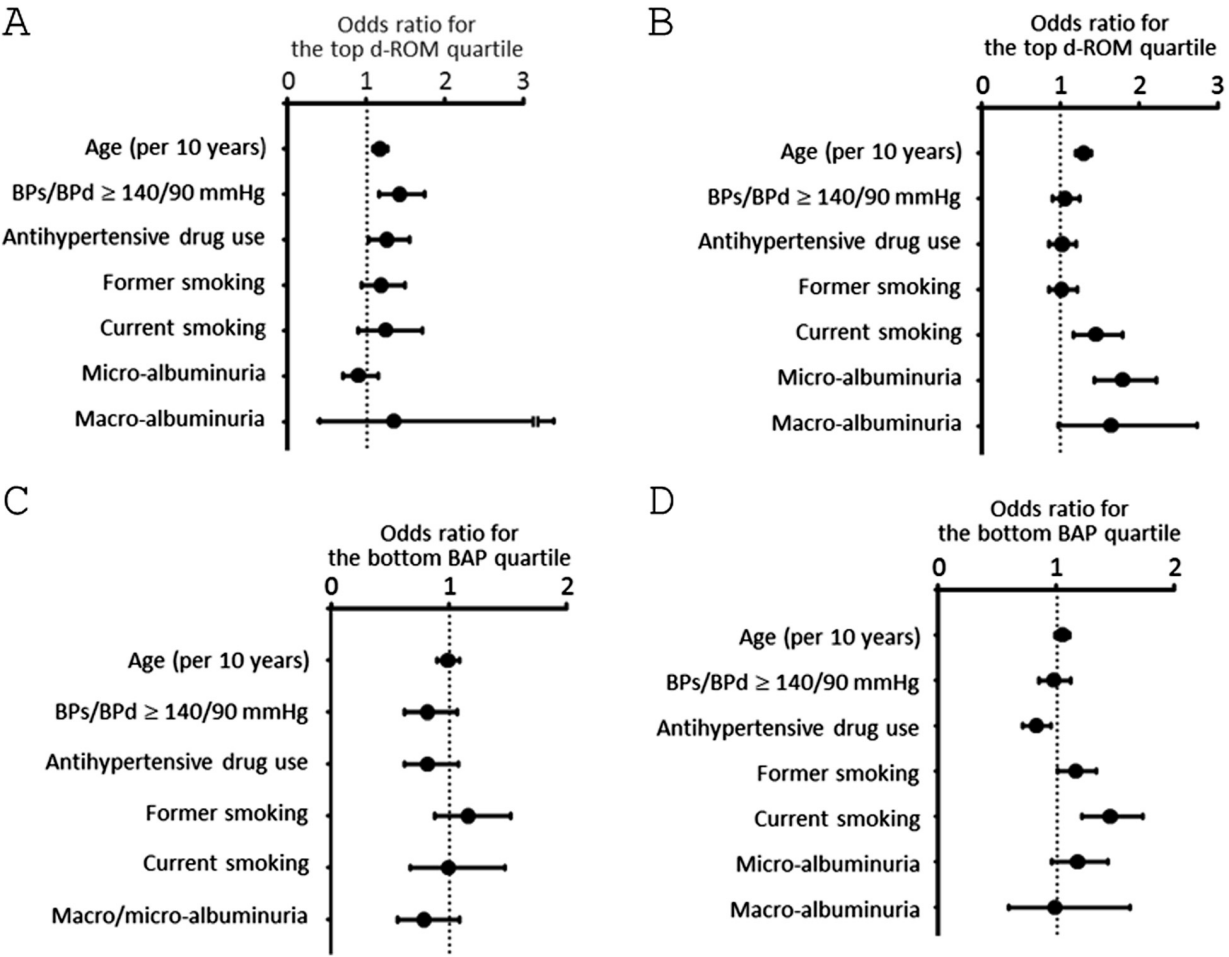
**Logistic regression analysis examining the association between micro-and macro-albuminuria and the top d-ROM and the bottom BAP quartile. A**. Association of various parameters with the top d-ROM quartile in women. **B**. Association of various parameters with the top d-ROM quartile in men. **C**. Association of various parameters with the bottom BAP quartile in women. Because of the low prevalence of macro-albuminuria in women, both micro-and macro-albuminuria was combined for the statistical analysis. **D**. Association of various parameters with bottom BAP quartile in men. For former and current smoking, never smoking was used as reference. For micro-and macro-albuminuria, normo-albuminuria (UAER < 30 mg/g·creatinine) was used as reference.

Next, we analyzed the whether albuminuria or low eGFR was associated with the top d-ROM quartile or with the bottom BAP quartile (Figure 5). After adjusting for age, high blood pressure, antihypertensive drug use, and smoking status, micro-and macro-albuminuria was associated significantly and borderline significantly, respectively, with the top d-ROM quartile in men, when normo-albuminuria (UAER < 30 mg/g·creatinine) was used as reference. After adjusting for the same variables, low eGFR (eGFR < 60 mL/min/1.73 m^2^) was not significantly associated with the top d-ROM quartile in women (odds ratio, 0.86, 95% CI 0.67-1.10) or in men (odds ratio, 0.91, 95% CI 0.74-1.13) when eGFR ≥ 60 mL/min/1.73 m^2^ was used as reference. Similarly, low eGFR (eGFR < 60 mL/min/1.73 m^2^) was not significantly associated with the bottom BAP quartile in women (odds ratio, 0.89, 95% CI 0.65-1.23) or in men (odds ratio, 0.84, 95% CI 0.70-1.01).

## Discussion

We found that, when the lowest UAER quartile (< 3.3 mg/g·creatinine) was used as reference, the higher two quartiles of UAER were positively associated with the top d-ROM quartile in both genders, and the higher three UAER quartiles were associated with the bottom BAP quartile in men, although the association between albuminuria and higher d-ROMs was weaken by the adjustment for CRP levels in women. These data indicate that increased UAER was associated with unfavorable oxidant status—high d-ROMs and/or low BAP—even in a range of normo-albuminuria (< 30 mg/g·creatinine). On the other hand, the first eGFR quartile (eGFR < 65.1 mL/min/1.73 m^2^) was associated, unexpectedly, negatively with the top d-ROM quartile in men.

### Relationship between eGFR, d-ROMs, and BAP levels

Several previous studies have analyzed the relationship between oxidative stress and renal function. By analyzing the data of community-dwelling men, Nerpin et al. showed that low eGFR was associated with the several inflammatory markers, such as CRP, interleukin 6 (IL-6) and serum amyloid A (SAA); however, low eGFR was associated with lower urinary F2-isoprostanes [23]. Similarly, Upadhyay et al. found in the Framingham Offspring Study that individuals with reduced renal function had lower urinary isoprostanes than those without [24]. These unexpected associations between reduced renal function and oxidant status might be owing to the fact that F2-isoprostanes were quantified not in plasma, but in urine [25]. On the other hand, however, Oberg et al. showed that plasma carbonyl or F2-isoprostaine did not closely correlate with eGFR [26]. By contrast, Rebholz et al. showed that patients with low eGFR had increased plasma fluorescent oxidation products, an oxidative stress marker that reflects oxidation products generated from several pathways including lipid, protein, DNA and carbohydrate oxidation [27]. Thus, it is possible that the type of biomarkers used may influence the result of the association of eGFR and oxidative stress.

Similar to the current study, Fukui et al. analyzed the relationship between serum creatinine and oxidant status markers in individuals undergoing general health screening, and found that subjects with higher serum creatinine, thus those with lower eGFR, had lower d-ROMs [12], which can be said to be comparable to the current study. Fukui et al. discussed that this may be attributed to the gender difference in d-ROM level. In the current study, however, negative association between the lowest eGFR quartile and the top d-ROM quartile was statistically significant in men, and borderline significant in women.

Low eGFR (< 60 mL/min/1.73 m^2^) was not found to be associated with the top d-ROM quartile in men, when eGFR of ≥ 60 mL/min/1.73 m^2^ was used as reference. On the other hand, the first eGFR quartile (< 65.1 mL/min/1.73 m^2^) was significantly negatively associated with the top d-ROM quartile when the fourth eGFR quartile (≥ 82.6 mL/min/1.73 m^2^) was used as reference (Figure 3).

### Relationship between UAER, d-ROMs, and BAP levels

Rashidi et al. found that proteinuria was associated with malondialdehyde levels, but not with oxidized low-density lipoproteins, in diabetic subjects [28]. In addition, Prior et al. showed that, in subjects with longstanding diabetes, plasma total antioxidant status did not differ according to the albuminuric status [29]. Furthermore, in the above-mentioned study, Nerpin et al. found that albuminuria was positively associated with several inflammatory markers including CRP, IL-6 and SAA; however, like reduced eGFR, increased albuminuria was associated with lower levels of urinary F2-isoprostanes in the study cohort [23]. As discussed above, this might be because urinary, but not plasma, F2-isoprostaines levels were measured in Nerpin et al.’s study. On the other hand, experimental studies showed albumin exposure will enhance the oxidative stress levels in the tubular cells. Souma et al. showed that oleic acid–bound albumin treatment may lead to an increased superoxide production in the proximal tubular cell line [30], and, in addition, Takao et al. showed that albumin treatment significantly increased 8-hydroxy-2′-deoxyguanosine levels in the culture media of the proximal tubular epithelial cells [31]. Therefore, it is possible that relationship between albuminuria and oxidative stress markers may, again, depend on the type of used biomarkers.

In the current study, it was found that, in men, either micro-or macro-albuminuria was associated with an increased odds for the bottom BAP quartile when normo-albuminuria was used as reference. It should also be noted that men with UAER of ≥ 3.3 mg/g·creatinine already had increased risk of having the bottom BAP quartile when compared with those with UAER of < 3.3 mg/g·creatinine (Figure 4B). In other words, men with UAER of ≥ 3.3 mg/g·creatinine, which is within normo-albuminuria range, already had increased risk for decreased BAP compared with those with UAER of < 3.3 mg/g·creatinine. We may have to be aware of this point when analyzing the relationship between albuminuria and antioxidant potential levels.

It has been reported that d-ROM levels showed positive association with CRP levels [19]. We therefore added CRP as one the covariates for the statistical model (Table 3); however, essentially the same results were obtained even after the further adjustment for CRP, except for the association between UAER and d-ROMs in women.

The current study has some limitations. First, although anti-hypertensive drug use was found to be positively associated with higher BAP, we did not have data regarding the type of anti-hypertensive medication used. Second, information regarding whether individuals were taking any anti-oxidative supplements, such as vitamin C and E, was not available. Third, although measurement of the d-ROMs and BNP is feasible for the assessment of oxidant status in a large scale population, different association between renal parameters and oxidant status biomarkers may be obtained according to the types of oxidative stress measurement used [32]. Fourth, although the eGFR equation used was validated by assessing the population aged 18 years or older, it was derived from mostly from patients with chronic kidney disease [22]. Thus, eGFR calculation may have certain inaccuracy when applying essentially healthy subjects, as has been done in the current study.

## Conclusion

In summary, by analyzing data from individuals who underwent general health screening, we found that albuminuria was associated with increased oxidative stress in both genders and low antioxidant potential in men, and the association between albuminuria and higher d-ROMs may be weaken by the adjustment for CRP levels in women. On the other hand, low eGFR was associated positively with low BAP levels, whereas, unexpectedly, negatively with high d-ROMs in men. Whether life-style modification and drug intervention alter these oxidant status markers awaits further investigation.

## Competing interests

The authors declare that they have no competing interests.

## Authors’ contributions

YI participated in the design of the study. MY have been involved in drafting the manuscript or revising it critically for important intellectual content. MT made substantial contributions to acquisition and interpretation of data. AT made substantial contributions to acquisition and interpretation of data. NI performed the statistical analysis. All authors read and approved the final manuscript.

## Pre-publication history

The pre-publication history for this paper can be accessed here:

http://www.biomedcentral.com/1471-2369/14/191/prepub
